# Knockout of zebrafish interleukin 7 receptor (IL7R) by the CRISPR/Cas9 system delays retinal neurodevelopment

**DOI:** 10.1038/s41419-018-0337-z

**Published:** 2018-02-15

**Authors:** Shijiao Cai, Yang Chen, Yue Shang, Jianlin Cui, Zongjin Li, Yuhao Li

**Affiliations:** 1 Key Laboratory of Tumor Microenvironment and Neurovascular Regulation, Nankai University School of Medicine, Tianjin, 300071 China; 2 State Key Laboratory of Medicinal Chemical Biology and College of Pharmacy, Nankai University, Tianjin, 300350 China; 3 Medical International Collaborative Innovation Center, Nankai University, Tianjin, 300071 China

## Abstract

*Interleukin 7 receptor* (*il7r*), a transmembrane receptor, belongs to the type I cytokine receptor family. *Il7r* is involved in the pathogenesis of neurodegenerative disorders, such as multiple sclerosis. Targeted knockdown of *il7r* leads to delayed myelination, highlighting the potential role of *il7r* in the development of the nervous system. Zebrafish is an ideal model for the study of neurogenesis; moreover, the *il7r* gene is highly conserved between zebrafish and human. The aim of the present study was to investigate the novel function of *il7r* in neurogenesis. First, an *il7r* ^−/−^ homozygous mutant line was generated by clustered regularly interspaced short palindromic repeats (CRISPR)-associated 9 (CRISPR/Cas9) technology. Second, the gross development of *il7r*^−/−^ mutants revealed remarkably smaller eyes and delayed retinal neurodifferentiation. Third, microarray analysis revealed that genes associated with the phototransduction signalling pathway were strongly down-regulated in *il7r* ^−/−^ mutants. Finally, the results from behavioural tests indicated that visual function was impaired in *il7r* ^−/−^ mutant larvae. Overall, our data demonstrate that a lack of *il7r* retards the development of the retina. Thus, *il7r* is an essential molecule for maintaining normal retinal development in zebrafish.

## Introduction

Interleukin 7 (IL7) is a cytokine produced by bone marrow and stromal cells of the thymus^1^. The IL7 receptor (IL7R) is a heterodimer formed by the specific IL7R alpha chain (IL7Rα) and common gamma chain (γc), which is shared by other cytokine receptors, such as IL2, IL4, IL9, IL15, and IL21. When IL7 binds to IL7R, IL7R/IL7Rα-γc activates its downstream pathways, including JAK/STAT, PI3K/Akt/mTOR and SOS/Ras/ERK^2^. *Il7r* plays a vital role in maintaining the development and homoeostasis of T-lineage and B-lineage cells^3,4^. Lack of *il7r* contributes to some haematological or immunological disorders, such as T-cell acute lymphoblastic leukaemia and severe combined immunodeficiency^5,6^. In recent years, more attention has been paid to the correlation between *il7r* and neurodegenerative diseases. *Il7r* is involved in the pathogenesis of demyelination in both patients with multiple sclerosis (MS) and animal models of experimental autoimmune encephalomyelitis^7–10^. A previous study in our lab revealed that *il7r* is an essential molecule for myelination. Interestingly, temporary deficiency of *il7r* caused smaller eyes in a zebrafish model^11^. This finding highlights the importance of exploring the potential impact of *il7r* on neurogenesis.

In the past few decades, three revolutionary genome-editing techniques, including zinc finger nuclease, transcription activator-like effector nuclease and clustered regularly interspaced short palindromic repeats (CRISPR)-associated (CRISPR/Cas), have been developed^12–14^. CRISPR/Cas systems are adaptive immune systems that protect bacteria and archaea against invasive viruses and plasmid DNA and are considered a third-generation genome-editing tool^15,16^. The three types of CRISPR/Cas systems are type I, type II and type III^17^. Type II CRISPR/Cas9 is the simplest system and merely composed of endonuclease Cas9 and single-guide RNA (sgRNA). Cas9 is guided by sgRNA to mediate site-specific recognition and double-strand breaks. sgRNA is the only element required for each genomic target, thus dramatically simplifying design and increasing efficiency^18,19^. Therefore, the CRISPR/Cas9 system has been widely used as an effective and precise way to modify genes.

In this study, an *il7r*^−/−^ mutant zebrafish line was generated using the CRISPR/Cas9 system to investigate the effect of the *il7r* gene on retinal development. Based on this zebrafish model, we determined the following properties: (1) gross development and neuronal differentiation of *il7r*^−/−^ mutants; (2) gene expression profiles, gene ontology (GO) and signalling pathway analysis and possible mechanisms following *il7r* knockout; and (3) behavioural changes, including movement and visual activity of *il7r*^−/−^ mutants. Our study might help to broaden our view of the novel function of the *il7r* gene in neuronal development.

## Results

### CRISPR/Cas9 system generates a targeted mutation of *il7r*

**Fig. 1:**
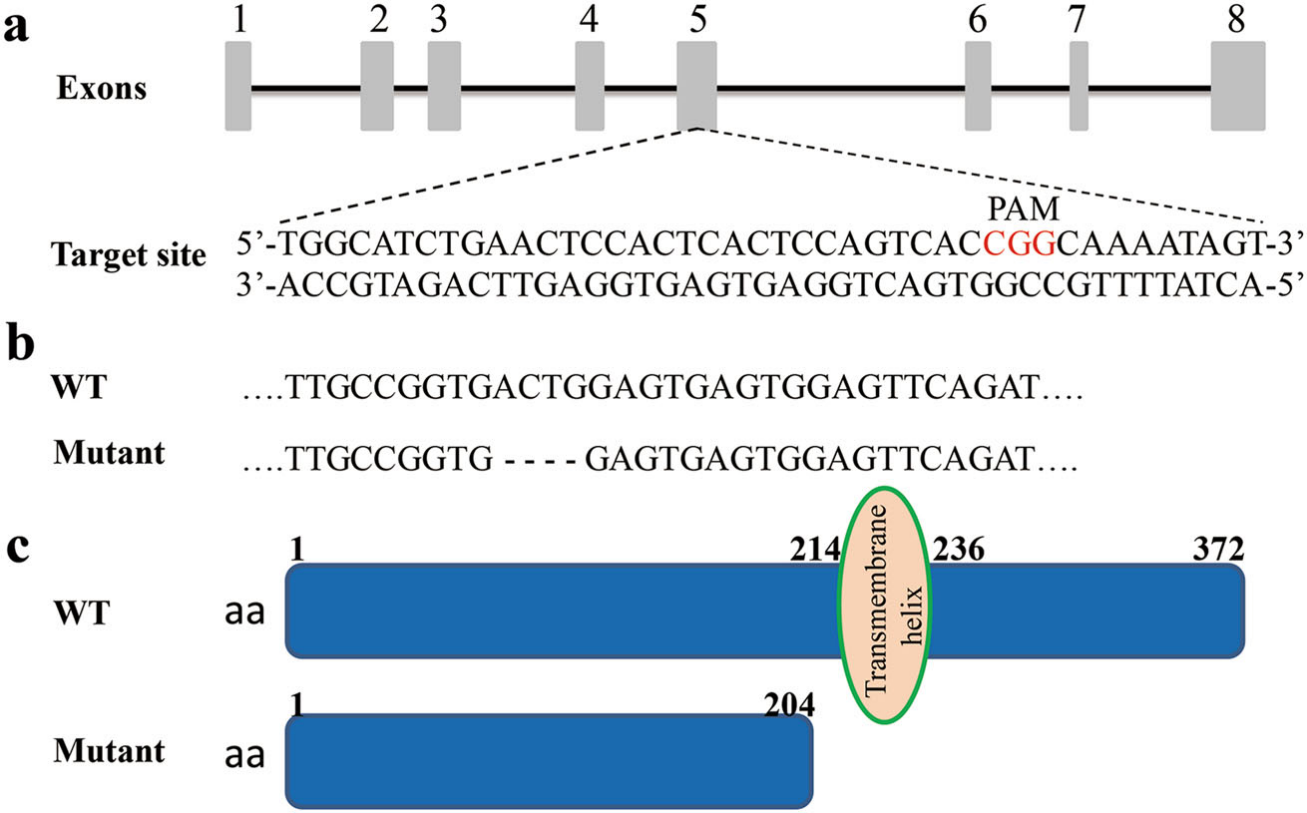
Generation of *il7r* mutant zebrafish with the CRISPR/Cas9 system. **a** Diagram of the target site in the zebrafish *il7r* genome. **b** Sequence alignment between WT and *il7r*^−/−^ mutant. **c** The predicted truncation of IL7R protein

**Fig. 2:**
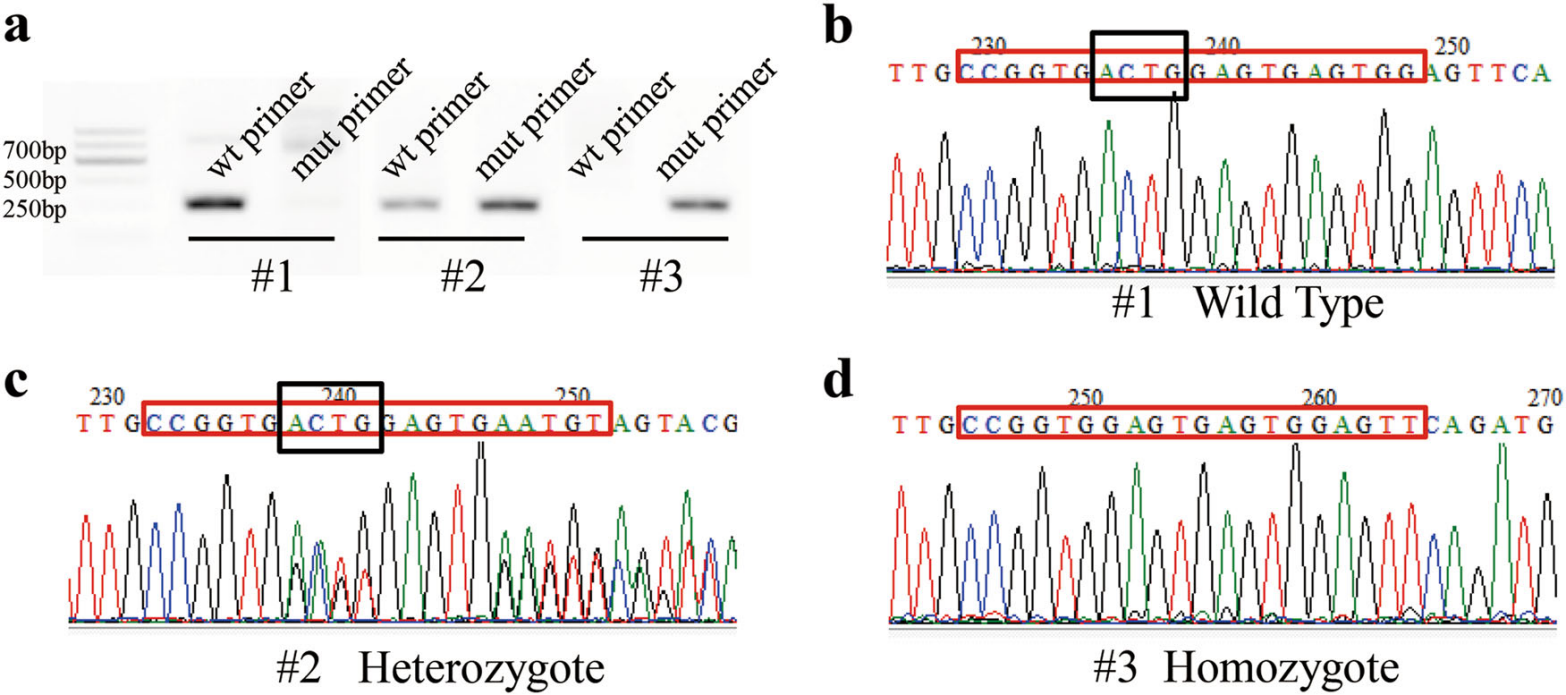
Identification of *il7r*^−/−^ homozygotes in F2 zebrafish. **a** PCR amplification of wild-type (#1), heterozygous (#2) and homozygous (#3) zebrafish. **b-d** Sequencing maps of wild-type (**b**), heterozygous (**c**) and homozygous (**d**) zebrafish. Red frames: sequences of the target site. Black frames: ACTG base pairs in wild-type and heterozygous zebrafish. Note the 4-bp (ACTG) deletion in homozygotes

In this study, we designed and generated a sgRNA targeting exon 5 of the *il7r* gene (Fig. 1a). F1 fish were generated by the self-cross of F0. We identified the genotype and found the deletion of four base pairs (bp) in F1 heterozygotes (Fig. 1b). This mutation led to a truncation of IL7R protein, which contained only 204 amino acids and lacked a transmembrane helix (Fig. 1c). The PCR results from wild-type (WT), heterozygous and homozygous larvae are shown in Fig. 2a. Compared with WT larvae (Fig. 2b), heterozygous larvae exhibited double peaks at the site of ACTG (Fig. 2c; black frame). However, homozygous larvae (Fig. 2d) showed a 4-bp deletion (site of ACTG, black frames in Fig. 2b, c). Moreover, there were no double peaks in the sequencing map, which was consistent with the PCR results. Since the CRISPR/Cas9 system has potential off-target effects, we analysed the top ten sites by a PCR assay and Sanger sequencing in homozygotes^20,21^. All the ten potential sites showed the same sequences as WT fish without double peaks (Supplementary Figure 1). Therefore, no detectable off-target effect was found in F2 *il7r*^−/−^ mutants.

**Fig. 3:**
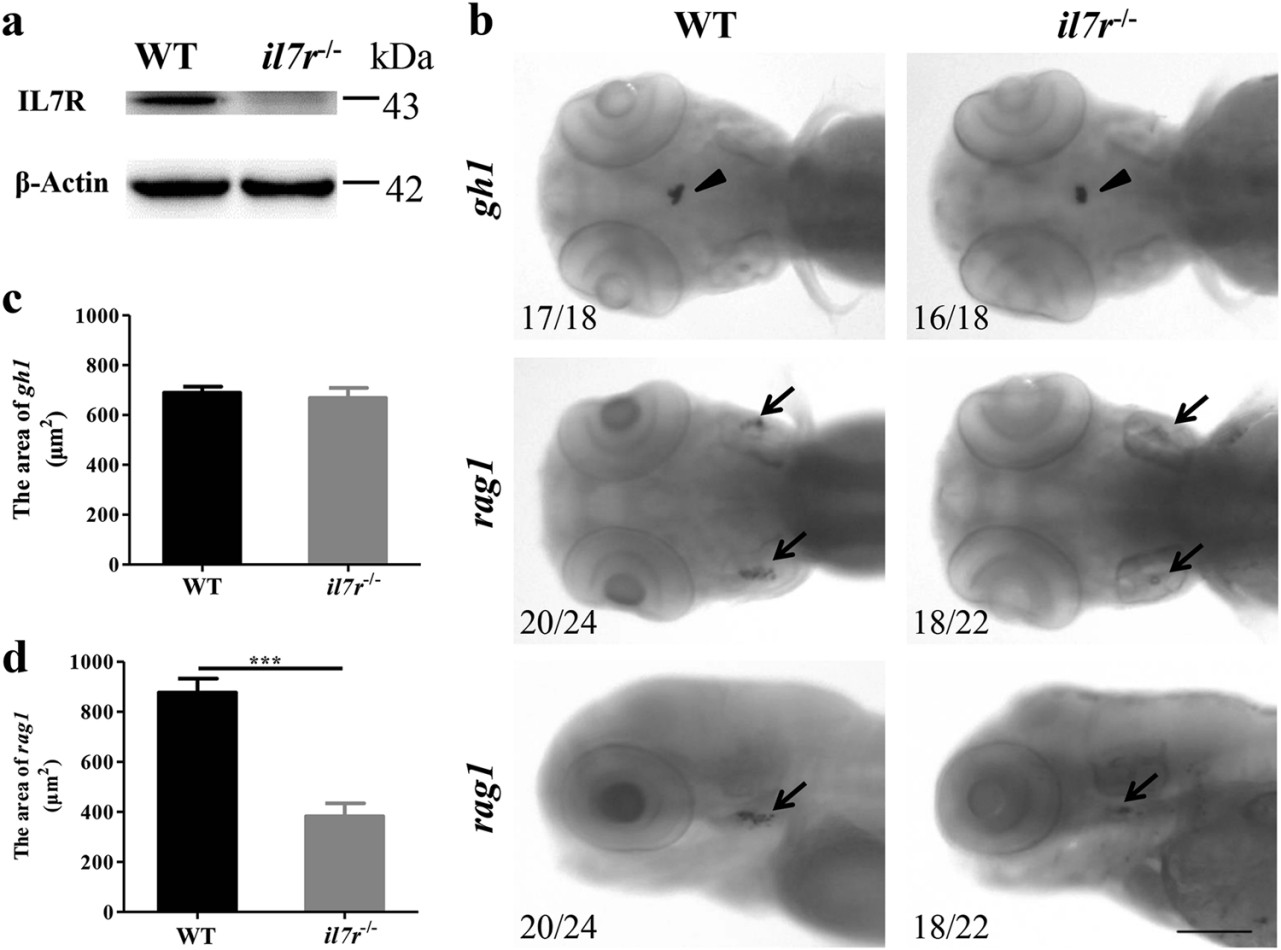
The validation of the *il7r*^−/−^ mutant by western blotting and whole-mount in situ hybridisation. **a** The expression of IL7R protein in WT and *il7r*^−/−^ larvae at 4 dpf. Note that no IL7R protein is detected in *il7r*^−/−^ larvae. **b** The images of whole-mount in situ hybridisation with *gh1* (arrowheads) and *rag1* (arrows) mRNA probes in WT and *il7r*^−/−^ larvae at 4 dpf. **c-d** The statistical analysis of *gh1*-positive area (**c**) or *rag1*-positive area (**d**) between WT and *il7r*^−/−^ larvae. Note that *rag1* expression is significantly decreased in *il7r*^−/−^ larvae. Results are represented as means ± SEM, ***P < 0.001. The upper four panels in **b**: dorsal view. The lower two panels in **b**: dorsal is up, and rostral is left. Scale bar in **b**: 100 μm

For the *il7r*^−/−^ mutant line, two approaches were used to verify reliability. First, we examined the expression of IL7R protein by western blotting. No IL7R protein was detected in *il7r*^−/−^ larvae (Fig. 3a). Second, we performed whole-mount in situ hybridisation at 4 days post fertilisation (dpf) with *growth hormone 1* (*gh1*) and *recombination activating gene 1* (*rag1*) mRNA probes, which label the hypophysis and thymus, respectively^22^. In zebrafish, *il7r* deficiency leads to the absence of lymphocytes in the thymus rather than in the hypophysis^3^. *Gh1* signals were detected in the hypophysis from both WT and *il7r*^−/−^ larvae (Fig. 3b, arrowheads), and no significant difference was found in the area of *gh1*-positive signals between the two groups (Fig. 3c; Student’s *t* test, *P* > 0.05). By contrast, only weak *rag1*-positive signals were detected in the bilateral thymic anlage of *il7r*^−/−^ larvae (Fig. 3b, arrows), although the cellular localisation of *rag1-*positive signals was similar between WT and *il7r*^−/−^ larvae. The area of *rag1*-positive signals in *il7r*^−/−^ larvae was dramatically lower than that in WT larvae (Fig. 3d; Student’s *t* test, ***P < 0.001), indicating that thymopoiesis was severely impaired after *il7r* knockout. Taken together, these results suggested that an *il7r*^−/−^ mutant line was successfully generated with the CRISPR/Cas9 system.

### Retinal development is delayed following *il7r* knockout

**Fig. 4:**
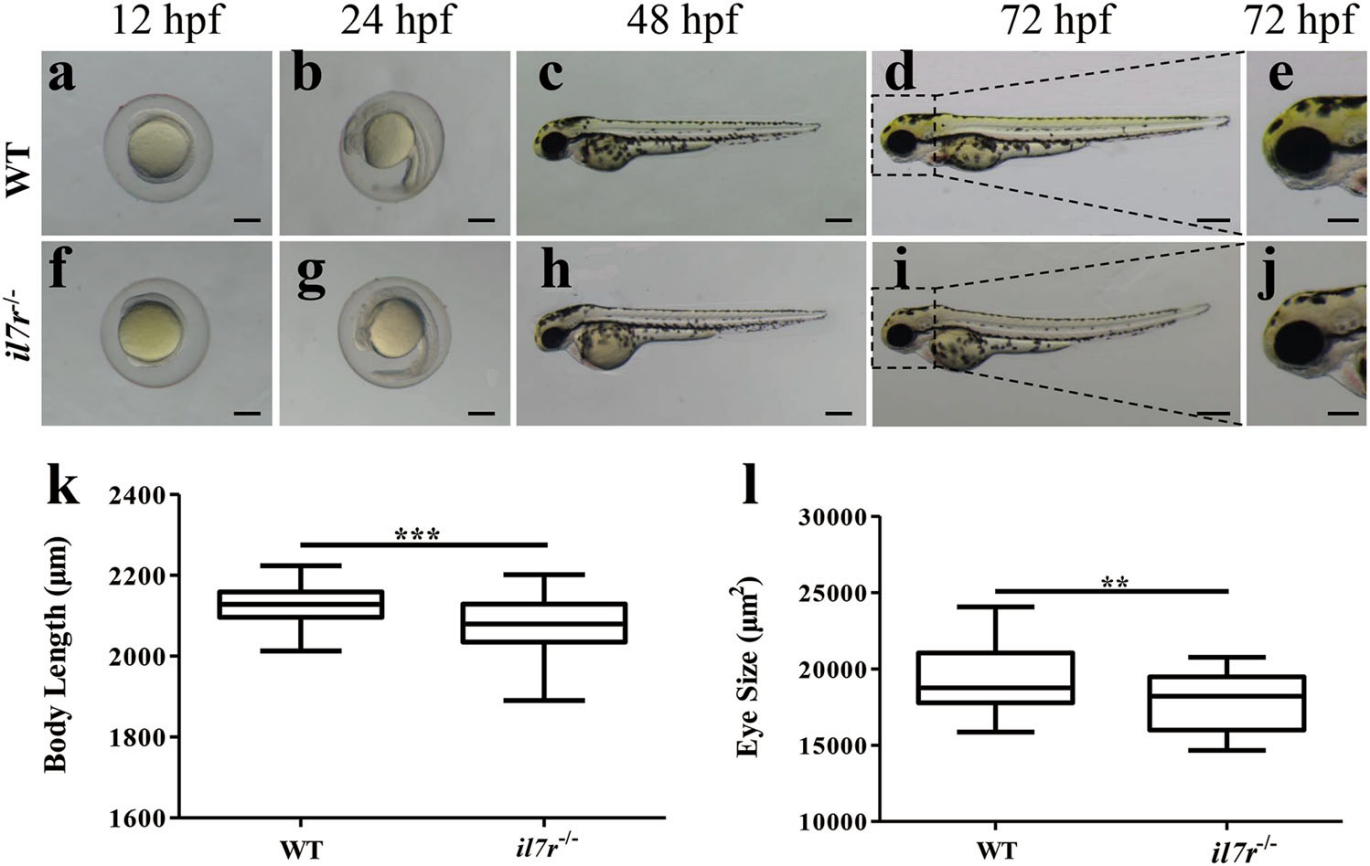
The phenotypes of the *il7r*^−/−^ mutant. **a-j** Phenotypes of WT and *il7r*^−/−^ embryos at 12 hpf (**a** and **f**), 24 hpf (**b** and **g**), 48 hpf (**c** and **h**) and 72 hpf (**d** and **i**). **e** and **j** are the magnified images of the heads from **d** and **i**, respectively. **k** Statistical analysis of body length in WT and *il7r*^−/−^ embryos at 72 hpf. Note that the body length of embryos in the *il7r*^−/−^ group is shorter than that embryos in the WT group, results are represented as means ± SEM (*n* = 30), ***P < 0.001. **l** Statistical analysis of eye size between the two groups. Note that the *il7r*^−/−^ mutants have smaller eyes, results are represented as means ± SEM (*n* = 30), **P < 0.01. Dorsal is up, and rostral is left in **c**, **d**, **e**, **h**, **i** and **j**. Scale bar: **a-d** and **f-i**, 200 μm; **e** and **j**, 100 μm

The phenotypes of WT and *il7r*^−/−^ larvae were assessed at 12 h post fertilisation (hpf), 24 hpf, 48 hpf and 72 hpf. Compared with WT embryos (Fig. 4a–c), *il7r*^−/−^ embryos showed no apparent malformation (Fig. 4f–h) before 48 hpf. However, compared with WT embryos (Fig. 4d, e), *il7r*^−/−^ embryos exhibited shorter body length and smaller eyes at 72 hpf (Fig. 4i, j). We then quantified these findings in embryos from the WT and *il7r*^−/−^ groups at 72 hpf (*n* = 30 in each group). *Il7r*^−/−^ larvae showed remarkable reductions in body length and eye size (Fig. 4k, l; Student’s *t* test, **P < 0.01, ***P < 0.001).

**Fig. 5:**
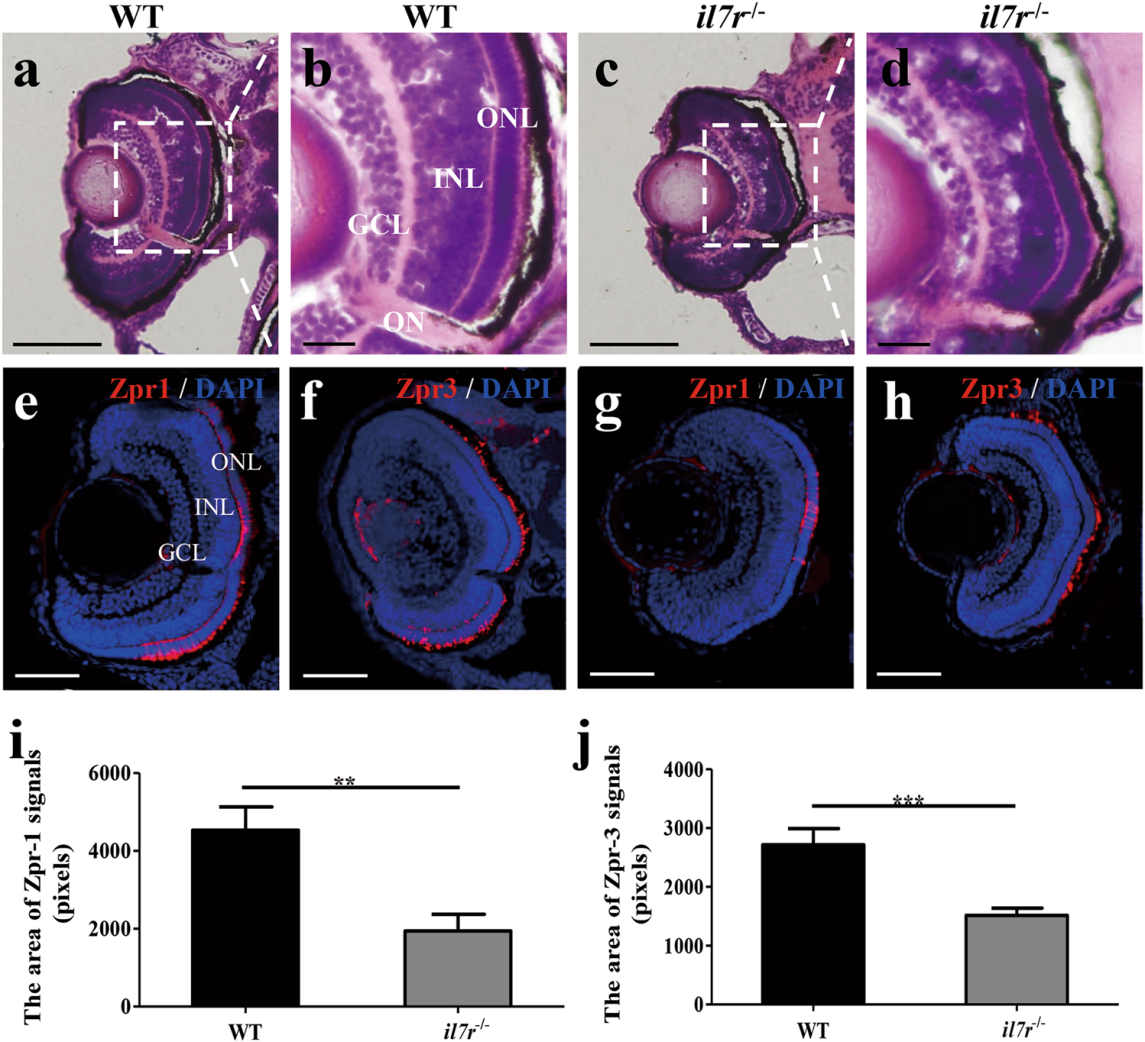
Retinal development following *il7r* knockout. **a-d** HE staining and magnified images of retinas from wild-type (WT, **a** and **b**) and *il7r*^−/−^ (**c** and **d**) embryos at 72 hpf. **e**–**h** Images of Zpr1 or Zpr3 immunofluorescence staining of retinas in WT (**e** and **f**) and *il7r*^−/−^ (**g** and **h**) embryos at 72 hpf. **i**-**j** Statistical analysis of Zpr1-positive signals (**i**) and Zpr3-positive signals (**j**) between WT and *il7r*^−/−^ retinas. Note that the Zpr1-positive area (**i**) and Zpr3-positive area (**j**) are significantly decreased in *il7r*^−/−^ retinas. Results are represented as means ± SEM (*n* = 10), **P < 0.01, ***P < 0.001. Scale bar: **a** and **c**, 40 μm; **b** and **d**, 10 μm; **e**–**h**, 50 μm. GCL ganglion cell layer, INL inner nuclear layer, ONL outer nuclear layer, ON optic nerve

To investigate whether smaller eyes were associated with abnormal retinal development, we performed haematoxylin and eosin (HE) staining on cross sections through the optic nerve of the retina. At 60 hpf, retinal cells began to laminate in both WT and *il7r*^−/−^ groups (Supplementary Figure 2a–d). At 72 hpf, cells in the retina from the WT group were well laminated, and the ganglion cell layer (GCL), inner nuclear layer (INL) and outer nuclear layer (ONL) were distinct (Fig. 5a, b). However, cells in the retina from the *il7r*^−/−^ group were smaller than those in the retina from the WT group (Fig. 5c). Furthermore, the GCL, INL and ONL from the *il7r*^−/−^ group were thinner than those from the WT group. Compared with nuclei in the WT group, nuclei in the *il7r*^−/−^ group were dense and darkly stained, with an increased nuclear-to-cytoplasm ratio (Fig. 5d). We then performed immunohistochemistry to explore neuronal differentiation in the retina at 60 hpf and 72 hpf, respectively. Zpr1 antibody can combine with protein encoded by *arrestin3a* (*arr3a*), which is specifically expressed in green and red double cones, while Zpr3 antibody binds to protein encoded by *rhodopsin* (*rho*) in rods. At 60 hpf, Zpr1-positive and Zpr3-positive signals distributed segmentally along the ONL of retina in WT group (Supplementary Figure 2e and f). However, Zpr1-positive and Zpr3-positive signals were weak and sporadic in *il7r*^−/−^ group (Supplementary Figure 2g and h). Statistical analysis showed that the area of Zpr1-positive or Zpr3-positive signals were fewer in the *il7r*^−/−^ group than those in the WT group (Supplementary Figure 2i and j; Student’s *t* test, ***P < 0.001). At 72 hpf, images from the WT group showed strong Zpr1-positive and Zpr3-positive signals distributed along the ONL, which indicated that cones and rods were well differentiated (Fig. 5e, f). The Zpr1-positive and Zpr3-positive signals in *il7r*^−/−^ embryos were also located in the ONL; however, the signals were sporadic (Fig. 5g, h), indicating that the retina was immature. To quantify this finding, we performed statistical analysis on the area of Zpr1-positive and Zpr3-positive signals between the WT and *il7r*^−/−^ groups. There was a significant decrease in these signals in the *il7r*^−/−^ group (Fig. 5i, j; Student’s *t* test, **P < 0.01, ***P < 0.001). The results from HE staining and immunohistochemistry showed that *il7r* knockout did not alter the location of retinal neurons but delayed eye development and neuronal differentiation of the retina.

### Genes related to phototransduction are down-regulated following *il7r* knockout

**Fig. 6:**
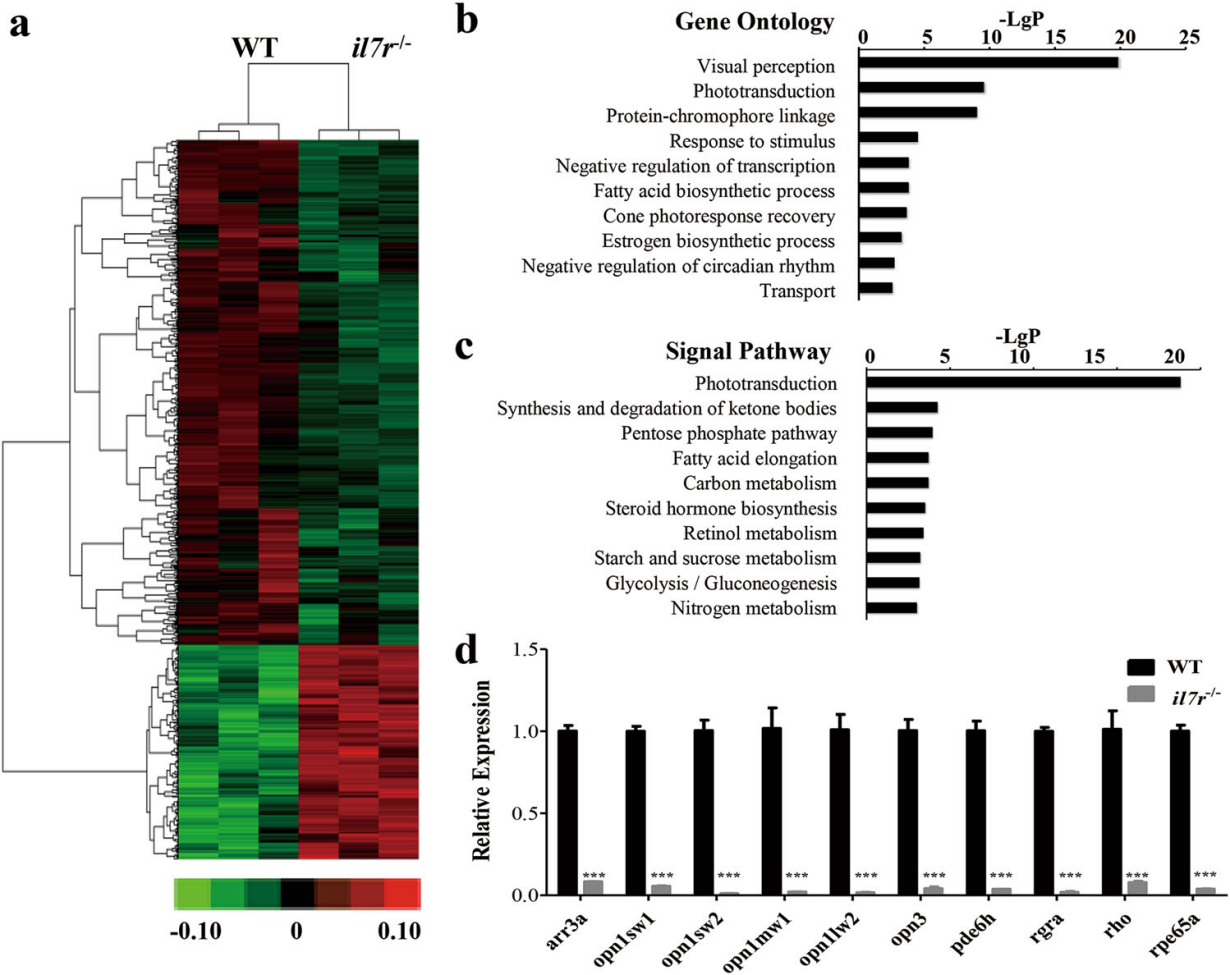
Differential gene expression profile, gene ontology (GO) and signalling pathway analysis following *il7r* knockout. **a** The heat map of the differential gene expression profile following *il7r* knockout assessed by cDNA microarray. **b** and **c** The analysis of the top ten down-regulated GO (**b**) and signalling pathways (**c**). **d** Ten genes related to visual perception and phototransduction were selected to validate the microarray results. Results are represented as means ± SEM, ***P < 0.001. -Lg *P*: negative logarithm value of *P* values from differential gene expression by microarray

To explore the possible mechanisms of delayed neurodifferentiation following *il7r* knockout, we performed microarray analysis using RNA isolated from WT and *il7r*^/−^ homozygotes at 4 dpf. A total of 1621 genes showed significant changes in gene expression profiles; 689 genes were up-regulated, and 932 genes were down-regulated (Fig. 6a). There were 64 down-regulated GO clusters, and the top ten GO clusters are listed in Fig. 6b. Visual perception and phototransduction were the first and second significantly decreased GO clusters following *il7r* knockout. We also performed signalling pathway analysis and detected ten relative down-regulated pathways (Fig. 6c). Interestingly, the phototransduction pathway also showed decreased expression. Based on GO and signalling pathway analyses, ten phototransduction-related genes were selected as follows: *arr3a*, *opn1sw1*, *opn1sw2*, *opn1mw1*, *opn1lw2*, *opn3*, *pde6h*, *rgra*, *rho and rpe65a*. We verified their expression by qRT-PCR. The expression of all 10 genes in the *il7r*^−/−^ group was significantly lower than that in the WT group (Fig. 6d; Student’s *t* test, ***P < 0.001). The above results illustrated that *il7r* knockout leads to inhibition of genes related to phototransduction.

### *Il7r*^−/−^ larvae exhibit weak responses to light stimulus

**Fig. 7:**
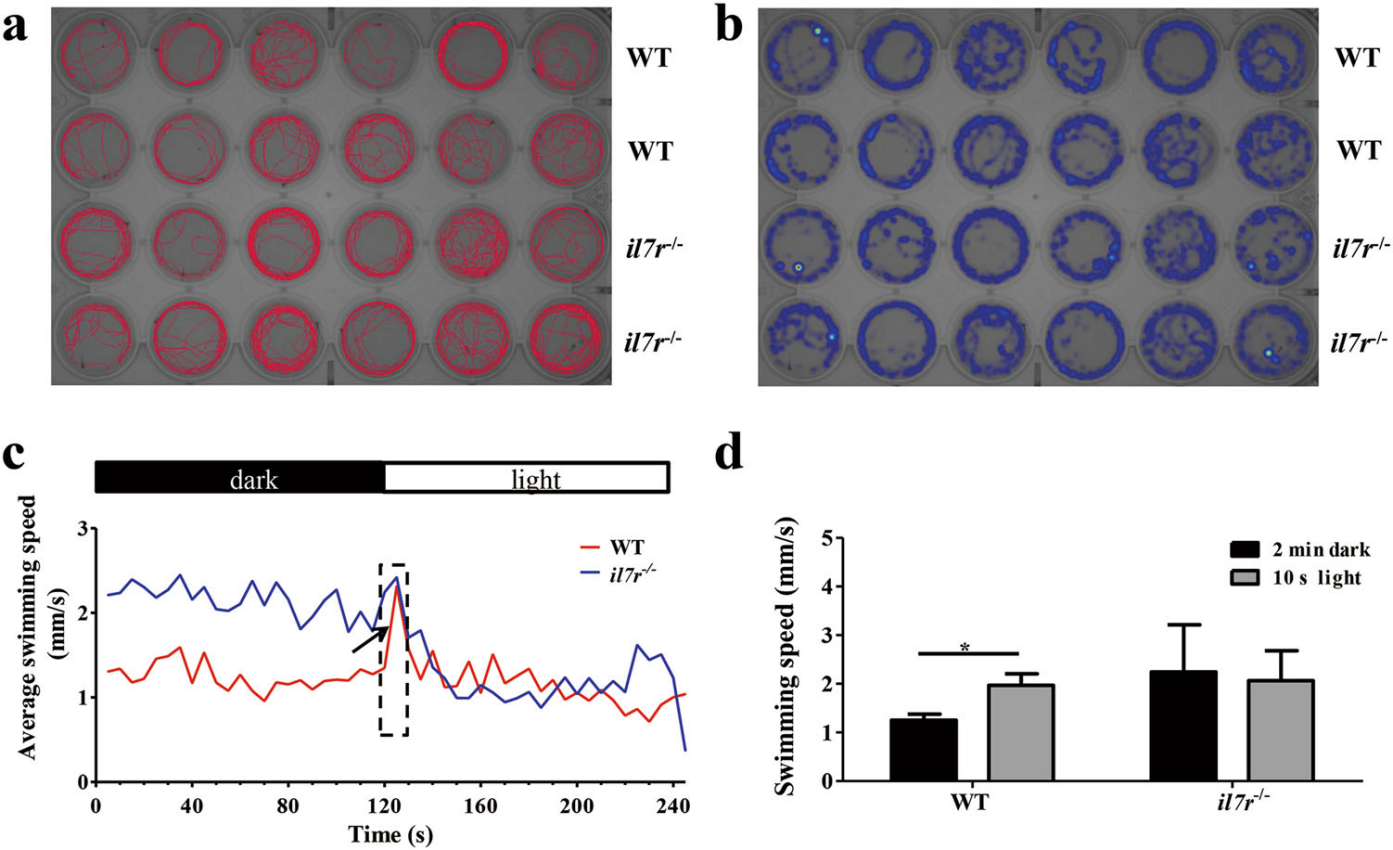
The swimming pattern and light response of *il7r*^−/−^ larvae. **a** and **b** The digital tracks (**a**) and corresponding heat maps (**b**) of larvae from the WT and *il7r*^−/−^ groups at 6 dpf. **c** The swimming speed in larvae from WT and *il7r*^−/−^ during the 2-min dark and 2-min light periods. Note that WT larvae exhibit a sharp increase in swimming speed in the first 10 s of the 2-min light period (arrow in dotted frame). **d** The statistical analysis of swimming speed during the 2-min dark period and the first 10 s of the 2-min light period in WT and *il7r*^−/−^ larvae. Note the increase in larvae from the WT group during the first 10 s of the 2-min light period. Results are represented as means ± SEM (*n* = 12), *P < 0.05

To investigate the functional changes following delayed retinal development, we collected larvae from the WT and *il7r*^−/−^ groups and performed behavioural testing at 6 dpf. The conditions included 2 min of darkness followed by 2 min of light. Digital tracks and heat maps were shown in Fig. 7a, b. When larvae were immediately exposed to light, WT larvae exhibited a sharp and upward peak in the first 10 s of 2 min of light (Fig. 7c, arrow in dotted frame). Compared with WT larvae, *il7r*^−/−^ larvae swam faster in the dark, and no sharp peak was observed in the first 10 s of the 2-min light period (Fig. 7c). We then analysed the velocities during 2 min of darkness and the first 10 s of light. The average speed was significantly increased after exposure to the light stimulus in WT larvae (Fig. 7d; Student’s *t* test, *P < 0.05). However, no significant difference was found between 2 min of darkness and 10 s of light in *il7r*^−/−^ larvae (Fig. 7d). These data indicated that *il7r*^−/−^ larvae had a weak response to light.

## Discussion

The main conclusions of this study are as follows: (1) an *il7r*^−/−^ mutant line was successfully generated using the CRISPR/Cas9 system and provides a model to investigate the novel function of the *il7r* gene; (2) *il7r* plays a regulatory role in neurogenesis, and its deficiency retards neuronal differentiation in zebrafish retina; and (3) *il7r* knockout down-regulates genes in the phototransduction pathway and further impairs visual function.

In this study, we used the CRISPR/Cas9 system to knockout the *il7r* gene in a zebrafish model. The activity of different sgRNAs varies based on different target genes; the general rate is in the range of 20–60%^18^. We designed a sgRNA targeting exon 5 and determined that the activity in our system was approximately 20%. We performed genotyping in F1 and found that the mutation was a 4-bp deletion. The ratio of F2 offspring was roughly consistent with Mendelian genetics. The off-target effect of CRISPR/Cas9 technology is a major concern due to mismatch recognition between sgRNA and the target sequence^20^. Several methods, including the Surveyor assay, T7E1 assay and Sanger sequencing were used to analyse the off-target effect^23^. We chose Sanger sequencing because of its efficiency and accuracy^21^. We confirmed that there was no off-target effect in the *il7r*^−/−^ mutant line. The 4-bp deletion in exon 5 led to a frameshift mutation in *il7r*, resulting in truncated IL7R protein, which caused the loss of the conservative function domain (transmembrane helix). Hence, we used an anti-IL7R antibody raised against a peptide at the C-terminus to verify reliability. The expression of IL7R was undetectable in the *il7r*^−/−^ mutant line. For further validation, we examined the development of the hypophysis and thymus by whole-mount in situ hybridisation. In *il7r*^−/−^ mutants, the thymus was severely underdeveloped, whereas the hypothesis was normal, which phenocopied the *il7r* morpholino-based knockdown that we previously reported^11^. Therefore, we successfully established an *il7r*^−/−^ mutant line. To our knowledge, this study is the first report of an *il7r* knockout zebrafish model. This mutant line generated by the CRISPR/Cas9 technique was stable and heritable. Furthermore, because the *il7r* gene is conserved between zebrafish and mammals, this knockout model is a powerful tool for in-depth investigation of the functions of the *il7r* gene as well as the mechanisms of *il7r*-related diseases.

The retina is considered an extension of the central nervous system in both anatomy and development^24^. The structure of the retina is highly conserved among vertebrates and consists of three distinct nuclear layers, the GCL, INL and ONL, separated by two plexiform layers^25^. The zebrafish retina is an excellent animal model to investigate neurogenesis and mechanisms of retinal diseases due to the retina’s spatial-temporal pattern of development. Photoreceptors, including cones and rods, play a vital role in phototransduction within the visual system^26^. Under physiological conditions, photoreceptors, including rods and cones, differentiate from 50 hpf. The first photoreceptors possess outer segments at 60 hpf within the ventral patch^27,28^. Photoreceptors are fully differentiated by 72 hpf^29^. Therefore, we chose 60 hpf and 72 hpf as time points to evaluate the retinal neurogenesis in the present study. The *il7r*^−/−^ mutant showed microphthalmia in gross development. By HE staining, we found thinning of the GCL, INL and ONL at 72 hpf. Furthermore, cells in the ONL were smaller and irregularly shaped with darkly stained nuclei. Immunohistochemistry results indicated that the number of both cones and rods was lower in *il7r*^−/−^ mutants either at 60 hpf or 72 hpf. Following *il7r* knockout, neurogenesis in the zebrafish retina was delayed. Our findings regarding the correlation between the *il7r* gene and retinal neurogenesis provide new insights into the neurogenic role of *il7r*.

We performed microarray analysis to reveal the possible mechanisms of delayed retinal development. Following *il7r* knockout, photoreceptor-specific genes were significantly down-regulated, and the phototransduction pathway was inhibited. Ten verified genes were classified into the following categories: (1) genes related to visual perception, such as *arr3a, rpe65a* and *pde6h*; (2) genes related to phototransduction, such as *rgra*, *opn1mw1* and *opn1sw1*; and (3) genes related to both visual perception and phototransduction, such as *rho*, *opn3*, *opn1lw2* and *opn1sw2*. The gene regulatory networks controlling photoreceptor specification include the following steps: retinal progenitors transforming into photoreceptors, rod versus cone fate, cone subtype, and opsin subtype^30^. In the first step, *retinal homeobox gene 1* (*rx1*), which belongs to the retinal homeobox family, contributes to photoreceptor fate^31^. *Rho*, a transmembrane protein located in rod cells, can initiate the visual transduction cascade when photo excited. *Rho* is closely associated with G-protein coupled receptor activity and photoreceptor activity. Furthermore, rhodopsin in rod photoreceptors can be phosphorylated and dephosphorylated to exert its function when exposed to photons^32^. *Arr3a*, a specific protein expressed in green and red cone photoreceptors, functions in phosphoprotein binding and opsin binding. In our microarray analysis, we found that the expression of *rx1*, *rho* and *arr3a* significantly decreased (Supplementary Figure 3a–c; ANOVA, *P < 0.05, **P < 0.01). We speculated that the possible mechanism underlying retinal development regulation by *il7r* was via the *jak1*, *jak3* or *stat5* pathway, which caused a decrease in the expression of *rx1* and its downstream genes *rho* and *arr3a* (Supplementary Figure 3d).

The following question emerged from the above findings: what are the functional changes following *il7r* knockout? The behavioural analysis of zebrafish larvae is a very intuitive and quick approach^33,34^. Zebrafish vision begins to function at 4 dpf^35^, and zebrafish larvae have the ability to swim at 5 dpf^36^. Therefore, we chose 6 dpf as the time point to test visual activity via monitoring swimming. Visual function was evaluated by a direct and non-invasive approach, which determined during movement 2-min dark and 2-min light periods^37^. Surprisingly, there was a big difference between *il7r*^−/−^ and WT larvae. In the dark, *il7r*^−/−^ mutants swam faster than WT larvae. We presumed that *il7r*^−/−^ caused anxiety. When larvae were suddenly stimulated with light, WT larvae had a robust reaction in the first 10 s. The average swimming speed increased by approximately 30%. However, *il7r*^−/−^ mutants hardly responded to light and showed a gradual decrease in swimming speed in the first 10 s. The swimming speed of WT and *il7r*^−/−^ mutants was similar in the light environment. Physiologically, when there is a light stimulus, zebrafish prefer to swim from the dark to the light; this phenomenon is called phototaxis^34^. We concluded that the weak response to the light stimulus indicated that the vision of *il7r*^−/−^ mutants was impaired. Moreover, the behavioural changes in *il7r*^−/−^ mutants were consistent with the morphological changes, including thinner layers of neurons, darkly stained nuclei, and fewer cones and rods.

Retinitis pigmentosa (RP), the most common inherited and degenerative retinal disease, causes severe vision impairment^38,39^. Usually, rods first undergo progressive degeneration, followed by deterioration of cones and retinal pigment epithelium^40^. There is no treatment or cure for RP. *Rho* and *arr3a* are involved in the pathogenesis of RP^38,41^. Our *il7r*^−/−^ model exhibited down-regulation of *rho* and *arr3a* with impaired visual function. Our findings indicate that this model may have applications for an in-depth understanding of the mechanisms and even the development of potential therapeutic approaches for RP.

Overall, the *il7r*^−/−^ knockout fish generated in this study is a powerful tool to investigate the roles of the *il7r* gene in vivo. Our study provides a new insight into the role of *il7r* in neurogenesis. This study will not only contribute to an understanding of the comprehensive functions of *il7r* but will also help elucidate the possible mechanisms in degenerative retinal diseases.

## Materials and methods

### Experimental animals

WT adult fish (Tübingen strain, TU) were raised at 28.5 °C under a 10/14-h dark/light cycle^42^. Embryos were collected after natural spawning and rinsed with system water to remove contaminants. Embryos were reared in E3 medium (5 mmol/L NaCl, 0.17 mmol/L KCl, 0.33 mmol/L CaCl_2_, and 0.33 mmol/L MgSO_4_, pH 7.2) in Petri dishes. Embryos/larvae were developmentally staged by hpf or dpf. All the animal experiments were approved by the Institutional Animal Care Committee of Nankai University and conformed to the National Institutes of Health Guidelines.

### CRISPR design, synthesis and microinjection

According to the principle of CRISPR/Cas9, sgRNAs against the *il7r* gene (ENSDARG00000078970) were designed using a CRISPR design tool (http://crispr.mit.edu/)^43^. The sgRNA target sequences for *il7r* were as follows: ACTCCACTCACTCCAGTCACCGG. sgRNA was generated with a pX330 vector template (BioVector NTCC Inc. Beijing, China) and transcribed using a MAXIscript T7 kit (Thermo Fisher Scientific, Waltham, MA, USA). The pGH-T7-zCas9 plasmid was linearised by *XbaI* and then transcribed in vitro to generate Cas9 mRNA using a mMESSAGE mMACHINE T7 kit (Thermo Fisher Scientific). Zebrafish embryos were injected with 1 nL mixed solution containing 50 ng/mL sgRNA and 250 ng/mL Cas9 mRNA^44^. Embryos were then incubated with sterile E3 medium and raised at 28.5 °C.

### Analysis of CRISPR-targeted mutation of *il7r*

At 7 hpf, 5 injected embryos were collected, and genomic DNA was extracted. A PCR assay was conducted to identify CRISPR-induced mutations. The screening primers were designed around the *il7r* sgRNA target site and amplified a 464-bp region in exon 5. The primer sequences were listed as follows: forward: *il7r*-exon5F: 5′-GGTTTGAACACCGTCATGATT-3′; reverse: *il7r*-exon5R: 5′-AAGTGGGATTTGAAACAACGA-3′. PCR products were cloned into the pGEM-T Easy vector (Promega, Madison, WI, USA). DNA isolated from a single colony was sequenced by *il7r*-exon 5 to verify the efficiency of the target site (the numbers of colonies with mutations/the total number of colonies sequenced).

### Generation of the *il7r*^−/−^ mutant line

At 1 month post fertilisation, the tail fins of the injected fish were cut and sequenced using an *il7r*-exon 5F primer to identify the F0 founder. F0 adults were self-crossed to generate F1 progeny. F1 embryos were raised normally until 60 dpf, and DNA of the caudal fin was extracted, PCR amplified and sequenced to identify heterozygous F1. F1 *il7r*^+/−^ heterozygotes were self-crossed to generate F2. F2 generation embryos were randomly selected, and genotype identification was performed at 4 dpf. Two forward primers, *il7r*-4 bp wt (sequence: 5′-AACTATTTTGCCGGTGAC-3′) and *il7r*-4 bp mut (sequence: 5′-AACTATTTTGCCGGTGGA-3′), and one common reverse primer, *il7r*-exon 5 R, were used to screen *il7r*^−/−^ homozygotes. To determine the off-target effects of the sgRNA:Cas 9 system, 50 potential sites were predicted among which 15 were identified in genes. The top ten sites were selected for further analysis by a PCR assay and sequencing^21^. The predicted sites and primers are listed in Table S1 and S2, respectively. A mutant line, *il7r* ^−/−^, was characterised and used for all data presented herein.

### Western blot analysis

At 4 dpf, 30 larvae from the WT or *il7r*^−/−^ groups were harvested and lysed with lysis buffer containing RIPA (CWBiotech, Beijing, China), phenylmethanesulfonyl fluoride (PMSF, Sigma, St. Louis, MO, USA), Protease Inhibitor Cocktail (PI, Promega), Phosphatase Inhibitor Cocktail 2 (PPI2, Sigma) and Phosphatase Inhibitor Cocktail 3 (PPI3, Sigma) at a ratio of 100:1:1:1:1. Western blot analysis was performed as previously described^11^. Anti-IL7R polyclonal antibody (1:400; Santa Cruz, sc-662, Dallas, TX, USA) was used as the primary antibody. Anti-actin monoclonal antibody (1:3000; Santa Cruz, sc-58679) was used as a loading control.

### Whole-mount in situ hybridisation

Embryos or larvae were treated with 0.003% 1-phenyl-2-thiourea (PTU, Sigma) to block pigmentation until 4 dpf. A standard protocol was used to perform whole-mount in situ hybridisation^45^. A *rag1* (GenBank NM_131389) mRNA probe was used as a marker to explore the development of the thymus. A *gh1* (GenBank NM_001020492) probe was used to label hormone-producing cells in the hypophysis. Probes were added to RNase free tubes at a concentration of 2 ng/μL and hybridised overnight at 55 °C. Larvae were washed and incubated with an alkaline phosphatase-conjugated antibody (Roche Diagnostics, Basel, Switzerland) at a dilution of 1:1500 on the second day. The colour reaction was mediated by nitro blue tetrazolium/5-bromo-4-chloro-3-indolyl-phosphate (NBT/BCIP, Roche) on the third day. The above experiment was repeated three times.

### Histology and immunohistochemistry

At 60 and 72 hpf, embryos were anaesthetised with 0.1% ethyl 3-aminobenzoate methanesulfonate salt (MS-222; Sigma), euthanized and immediately fixed in 4% paraformaldehyde. For histological analyses, larvae were embedded in paraffin, and serial transverse sections (5 µm) were obtained. HE staining was performed using a standard protocol. For immunohistochemistry, serial transverse cryosectioning (8 µm) was performed. The following primary antibodies were used in this study: mouse monoclonal antibodies Zpr1 (diluted at1:200, Zebrafish International Resource Center (ZIRC), Eugene, OR, USA) and Zpr3 (diluted at1:200, ZIRC) to label cone and rod cells, respectively. Ten embryos were processed in each group.

### Microarray analysis

At 4 dpf, 20 larvae from the WT and *il7r*^−/−^ groups were collected, anaesthetised and immediately euthanized. Total RNA was extracted, linearly amplified, labelled with a GeneChip^®^ WT Terminal Labelling and Controls Kit (Thermo Fisher Scientific) and quantified. The above experiment was repeated in three batches of larvae to generate biological replicates of WT and *il7r*^−/−^ samples. mRNA expression profiling was measured using a Zebrafish Gene 1.0 ST Array (Affymetrix, Santa Clara, CA, USA), which contains 59302 gene-level probe sets. GeneChip2 Scanner 30007 G (Affymetrix) was used to scan the hybridized arrays. Microarray analysis was performed by Expression Console Software (version 1.2.1; Affymetrix). The microarray data have been deposited in the National Center for Biotechnology Information Gene Expression Omnibus (GEO). The GEO series accession number is GSE101138 (https://www.ncbi.nlm.nih.gov/geo/query/acc.cgi?acc=GSE101138).

### Bioinformatics analysis

For microarray data analysis, differentially expressed genes were recognised based on one-way ANOVA. The criteria set for significantly different expression were identified as up-regulated or down-regulated according to the following standard: ANOVA *P*-value <0.05, |Fold change| >1.5. The false discovery rate was calculated to correct the *P*-value. GO analysis was performed to evaluate the affected biological processes and molecular functions. Two-sided Fisher’s exact test and multiple comparisons were used to classify the GO category, and a *P*-value <0.05 was considered statistically significant. Pathway analysis was applied to determine the significant pathways of the differentially expressed genes according to the Kyoto Encyclopedia of Genes and Genomes (KEGG) database. The significant pathway was selected by Fisher’s exact test. The threshold for KEGG significance was *P* < 0.05.

### Quantitative RT-PCR

At 4 dpf, 15 larvae were collected from the WT and *il7r*^−/−^ groups, and total RNA was isolated by using TRIzol reagent (Thermo Fisher Scientific) according to the manufacturer’s protocol. Quantitative RT-PCR (qRT-PCR) was performed using the *TransStart*^®^ Top Green qPCR Supermix (TransGen, Beijing, China). The procedures were as follows: 94 °C for 30 s, followed by 40 cycles of 94 °C for 5 s, and 60 °C for 30 s. The sequences of the primers are listed in Table 1. The relative expression of mRNA was calculated by the 2^−△△Ct^ method^46^. For each gene, the above experiment was performed on three independent duplicates.

**Table 1:** Primer sequences of genes related to visual perception and phototransduction for qRT-PCR

Gene	GeneBank	Sequence (5′ to 3′)
*arr3a*	NM_001002405	Forward: AAGACCTGGACGTGATTG
		Reverse: TTGAAAGTGAAGGGATGG
*opn1lw2*	NM_001002443	Forward: CAGCACAATCAGCGTCAT
		Reverse: TGCCCATTTACCATCAAA
*opn1mw1*	NM_131253	Forward: AGCCCAGCACAAGAAACT
		Reverse: AGCAACCTGACCTCCAAG
*opn1sw1*	NM_131319	Forward: GGCTTTGTATTTATCGTGG
		Reverse: CCTGCTAGGGAGATGTTTA
*opn1sw2*	NM_131192	Forward: GGACTCCCTCCACTCTTA
		Reverse: GAATACAATGGTGCTGAAA
*opn3*	NM_001111164	Forward: GAGAAGAAAGTGGCGGTGAT
		Reverse: ATAATGGCGACGGTAGGG
*pde6h*	NM_001305554	Forward: GACCACTCGCACCTTCAA
		Reverse: ATGTCTCCAAACGCTTCC
*rgra*	NM_001017877	Forward: GAGAAGAAAGTGGCGGTGAT
		Reverse: ATAATGGCGACGGTAGGG
*rho*	NM_131084	Forward: TCATCTGCTGGTTGCCCTA
		Reverse: CAGTGACGGAACTGCTTGTT
*rpe65a*	NM_200751	Forward: ACTCAACCATTTCGTCCCT
		Reverse: TACCGTCGTCCTCATCCA

### Behavioural test

The behavioural test was performed at 6 dpf and recorded with a DanioVision system (Noldus Information Technology, Wageningen, the Netherlands). Larvae were divided into the WT and *il7r*^−/−^ groups. Twelve larvae from each group were collected and placed in a 24-well plate. Each well contained one larva with 2 mL E3 medium. After a 30-min dark adaption in the chamber, the movement of larvae was recorded for 4 min, including a 2-min dark period followed by a 2-min light stimulus. Ethovision^®^ XT 11.5 software was used to analyse digital tracks and generate heat maps. The average velocities in the dark and in the first 10 s of light were set as the parameters to evaluate visual function. The behavioural test described above was repeated three times.

### Photography and image analysis

Images of the phenotypes of embryos or larvae and images of whole-mount in situ hybridisation were captured with a DP72 digital camera mounted on an SZX16 dissecting microscope (Olympus Corporation, Tokyo, Japan). DP2-BSW software (Olympus) was used to calculate the body lengths, eye sizes and areas of *gh1*/*rag1*-positive signals. Images of HE staining were photographed with a DP71 digital camera mounted on a BX51 fluorescence microscope (Olympus). Images of immunofluorescence were captured with an FV 1000 confocal microscope (Olympus). ImageJ software (1.49×; NIH, http://rsb.info.nih.gov/ij/) was used to convert the fluorescence images of Zpr1 or Zpr3 immunostaining to 8-bit greyscale prior to thresholding and calculate the area of positive areas in each image. All images were compiled in Adobe Photoshop CS6 Portable (Adobe Systems Incorporated, San Jose, CA, USA) and resized. All images involved in the experiment were similarly manipulated.

### Statistical analysis

Statistical analysis was performed with GraphPad software (version 5.01, GraphPad Software, Inc., La Jolla, CA, USA). All values are presented as the means ± SEM. Student’s *t* test was performed to evaluate statistical significance between two independent groups. Statistical significance was defined as a *P*-value less than 0.05.

## Electronic supplementary material

Supplemental data

Supplementary Figure 1

Supplementary Figure 2

Supplementary Figure 3
