# Supplementary material for: Knockout of zebrafish interleukin 7 receptor (IL7R) by the CRISPR/Cas9 system delays retinal neurodevelopment
Source: Cell Death Dis. 2018 Feb 15;9(3):273. doi: 10.1038/s41419-018-0337-z (PMC5833684; doi:10.1038/s41419-018-0337-z)
Supplement: Supplementary file 1 — Supplemental data [file 41419_2018_337_MOESM1_ESM.docx]

**Supplemental data**

Table S1. The top 10 potential off-target sites

| No. | Sequence (5’to 3’) | Mismatches | Locus | |
| --- | --- | --- | --- | --- |
| 1 | ACGCCACCCACTCCAGTCACTGG | 3MMs [3:8:11] | | chr11:-16561443 |
| 2 | ACTGCACACACTCCAGTCATAAG | 3MMs [4:8:20] | | chr1:+35409623 |
| 3 | ACTCCATTCACTCCATTCACTAG | 2MMs [7:16] | | chr16:+4061693 |
| 4 | CCCACACTGACTCCAGTCACTGG | 4MMs [1:3:4:9] | | chr21:+40257427 |
| 5 | CCACCATTTACTCCAGTCACTGG | 4MMs [1:3:7:9] | | chr17:+52090257 |
| 6 | ACACCATGCAATCCAGTCACCAG | 4MMs [3:7:11:8] | | chr2:+32172736 |
| 7 | TCTCCGCTATCTCCAGTCACTGG | 4MMs [1:6:9:10] | | chr21:+845404 |
| 8 | ACAGCACTAACTCCAGTCAGTAG | 4MMs [3:4:9:20] | | chr13:-40067934 |
| 9 | ACGCGCCTCACTCCAGTCAACGG | 4MMs [3:5:6:20] | | chr18:-17772404 |
| 10 | CCGCCACTCATTCCAGTCAATGG | 4MMs [1:3:11:20] | | chr3:-34309777 |

Table S2. Sequences of primers for detecting off-target effects

| No. | GenBank | Primer sequence (5’ to 3’) |
| --- | --- | --- |
| off-target1 | NM_00109941 | Forward: GATTCCACCGACTCTGTAA |
|  |  | Reverse: TTTATGTGCTCCACCTGTC |
| off-target2 |  | Forward: AAAAGTTCTCCAGGGTGTC |
|  |  | Reverse: AAGTGGCTTTGTCATTGCT |
| off-target3 | NM_001113377 | Forward: ATCCTGGAGACACCAAAATCTTA |
|  |  | Reverse: CGTGAACAACCAGCCAAACA |
| off-target4 | NM_00103012 | Forward: GGCTGATTTCTCACCTATCTCCT |
|  |  | Reverse: TTTCAGTGCTGAGTGGTGTGA |
| off-target5 |  | Forward: AAGTGGAAGATATGTTAGC |
|  |  | Reverse: ATTTTCTTACGACTGAACT |
| off-target6 |  | Forward: CCCGAGGTTATAGTCTTAA |
|  |  | Reverse: AAAATCTGCATAGGTCAAA |
| off-target7 |  | Forward: CAGCGTTAAAACCCAACT |
|  |  | Reverse: CTTCACATCTGTCCTCCC |
| off-target8 |  | Forward: CTGAACGTCACCTCACATG |
|  |  | Reverse: ATCAACCATCCGTGTCTTC |
| off-target9 |  | Forward: ACCTCAATTTTCCCCTCTA |
|  |  | Reverse: ATCTGGATTTATGTCTGCTT |
| off-target10 | NM_00104495 | Forward: AAACATCTGTGAATATCAAGCCTAC |
|  |  | Reverse: CACATTTGCCTCCCACTCT |

**Supplementary Figure 1** Detection of the off-target effects in F2 *il7r*^-/-^ homozygotes by Sanger sequencing. Red frames: sequences of the target site.

**Supplementary Figure 2** Retinal development following *il7r* knockout at 60 hpf. (**a-d**) HE staining and magnified images of retinas from wild-type (WT, **a** and **b**) and *il7r*^-/-^ (**c** and **d**) embryos. (**e**-**h**) Images of Zpr1 or Zpr3 immunofluorescence staining of retinas in WT (**e** and **f**) and *il7r*^-/-^ (**g** and **h**) embryos. (**i**-**j**) Statistical analysis of Zpr1-positive signals (**i**) and Zpr3-positive signals (**j**) between WT and *il7r*^-/-^ retinas. Note that the Zpr1-positive area (**i**) and Zpr3-positive area (**j**) are significantly decreased in *il7r*^-/-^ retinas. Results are represented as means ± SEM (n=10), ****P*<0.001. Scale bar: (**a** and **c**), 40 μm; (**b** and **d**), 10 μm; (**e**-**h**), 50 μm. Abbreviations: GCL: ganglion cell layer; INL: inner nuclear layer; ONL: outer nuclear layer.

**Supplementary Figure 3** The interaction between *il7r* and retinal development. (**a-c**) The changes in *rx1* (**a**), *rho* (**b**) and *arr3a* (**c**) across the microarray in the three replicates. Note that the expression of three genes are decreased significantly in *il7r*^-/-^ group (ANOVA; **P*<0.05, ***P*<0.01). (**d**) String-db analysis of *il7r*. Small nodes: proteins with unknown 3D structures; large nodes: proteins with known or predicted 3D structures.
