# Supplementary figures and images for: Knockout of zebrafish interleukin 7 receptor (IL7R) by the CRISPR/Cas9 system delays retinal neurodevelopment
Source: Cell Death Dis. 2018 Feb 15;9(3):273. doi: 10.1038/s41419-018-0337-z (PMC5833684; doi:10.1038/s41419-018-0337-z)

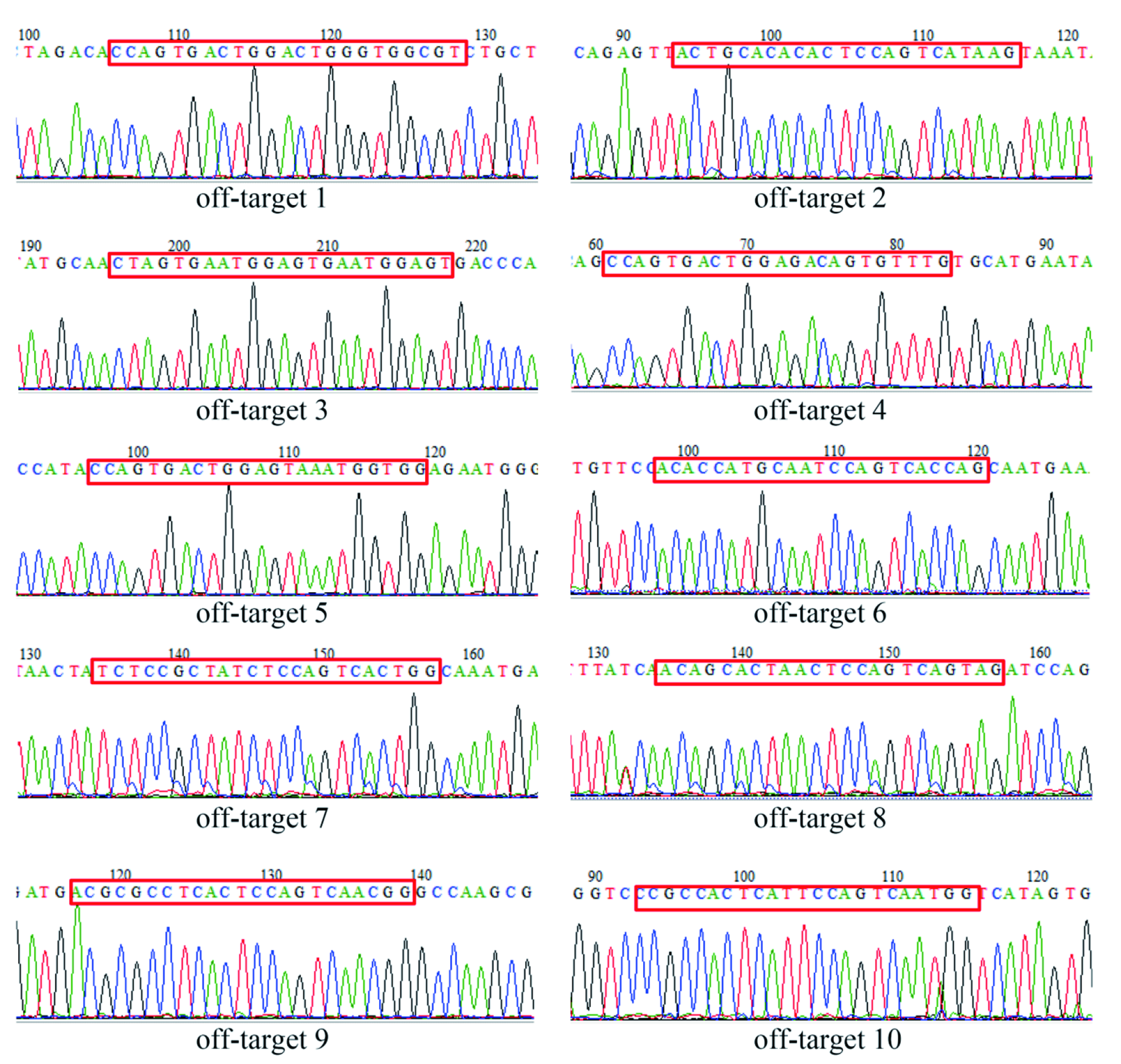

Supplement: Supplementary file 2 — Supplementary Figure 1 [file 41419_2018_337_MOESM2_ESM.tif]

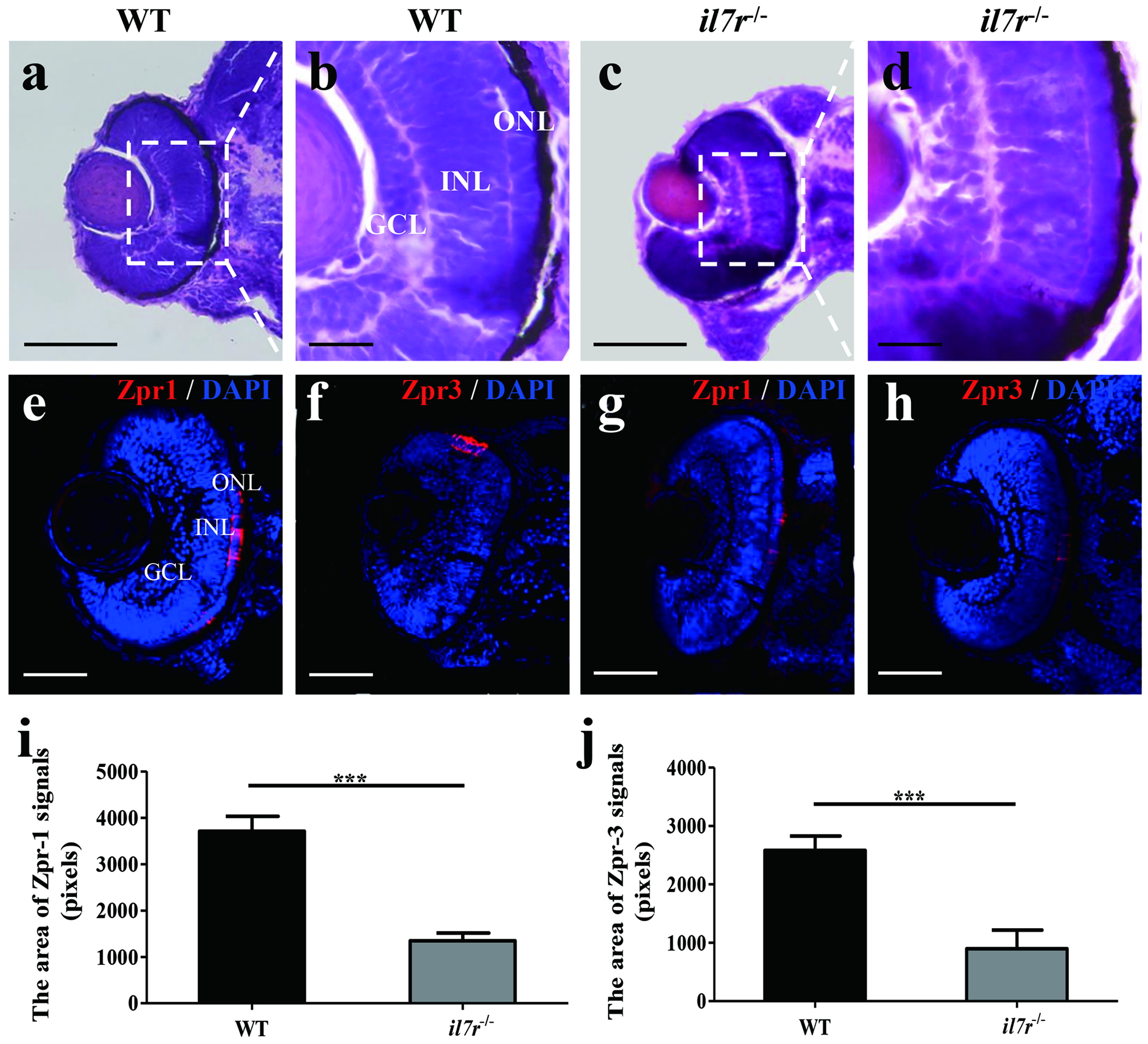

Supplement: Supplementary file 3 — Supplementary Figure 2 [file 41419_2018_337_MOESM3_ESM.tif]

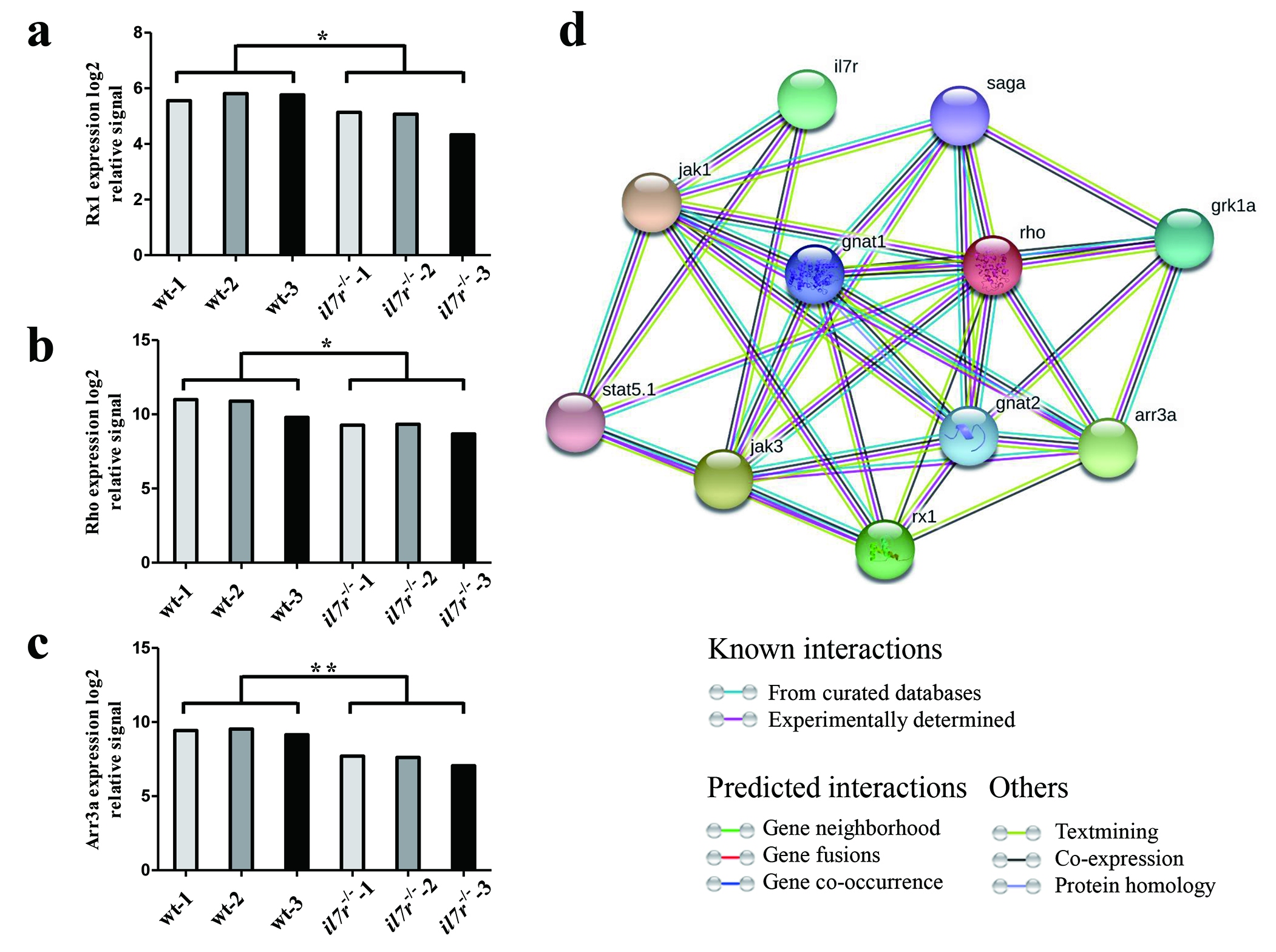

Supplement: Supplementary file 4 — Supplementary Figure 3 [file 41419_2018_337_MOESM4_ESM.tif]
